# Associations Between Patterns of Sleep Disturbances and Mental Health Among Child Welfare-Involved Adolescents

**DOI:** 10.3390/children13040441

**Published:** 2026-03-24

**Authors:** Camie A. Tomlinson, Tiarra Abell, Andreana Bridges, Becky Antle, Samantha M. Brown

**Affiliations:** 1 Kent School of Social Work and Family Science, University of Louisville, Louisville, KY 40292, USA; andreana.bridges@louisville.edu (A.B.); becky.antle@louisville.edu (B.A.); 2 Department of Psychological and Brain Sciences, University of Louisville, Louisville, KY 40208, USA; tiarra.abell@louisville.edu; 3 Department of Human Development and Family Studies, Colorado State University, Fort Collins, CO 80523, USA; samantha.brown@colostate.edu

**Keywords:** sleep disturbances, sleep health, mental health, childhood adversity, latent class analysis

## Abstract

**Background/Objectives**: Sleep is an important biobehavioral process that supports child and adolescent health and development. However, many prior studies examining sleep and mental health have relied on total sleep scores, which may mask the heterogeneity of sleep disturbances. Youth exposed to childhood adversity are at increased risk for sleep disturbances and poor mental health, and thus it is important to examine the links between sleep and mental health within adversity-exposed samples, such as those involved with the child welfare system. **Methods**: This study used latent class analysis to identify underlying patterns of sleep disturbances and examine differences in mental health symptoms (assessed at baseline and at an 18-month follow-up) across the identified subgroups in a sample of child welfare-involved adolescents (*N* = 1041, *M_age_* = 13.63 years, *SD* = 1.86). Our sample was derived from the second cohort of the National Survey on Child and Adolescent Well-Being (NSCAW) study. **Results**: We identified three subgroups of sleep disturbances: *no sleep disturbances* (38%), *sleeping more than peers and overtired* (16%), and *trouble maintaining sleep* (47%). We found significant mean differences in mental health symptoms across subgroups. Across internalizing, externalizing, and post-traumatic stress disorder (PTSD) symptoms at baseline and at an 18-month follow-up, those in the *no sleep disturbances* subgroup had overall lower levels of symptoms compared to those in the *trouble maintaining sleep* subgroup, which had higher levels of symptoms. Compared to those in the *sleeping more than peers and overtired* subgroup, the *trouble maintaining sleep* subgroup had higher levels of PTSD symptoms at baseline, and higher levels of externalizing and PTSD symptoms at the follow-up. Those in the *sleeping more than peers and overtired* subgroup had significantly higher levels of internalizing, externalizing, and PTSD symptoms at baseline compared to the *no sleep disturbances* subgroup, but there were no significant differences at the 18-month follow-up. **Conclusions**: The current study highlights the importance of considering the heterogeneity of sleep disturbances to identify child welfare-involved youth who may be more at risk for sleep disturbances and poor mental health and to inform more targeted sleep interventions for this population.

## 1. Introduction

Adolescence is a period of rapid neurological, physical, and social development. Exposure to adversity during this period can negatively influence these developmental changes. Sleep, which is often also disrupted in adolescence, plays a vital role in supporting healthy development [1,2]. Because it underpins emotional regulation, learning, and neurodevelopment, sleep may be an essential biological process linking childhood adversity to poor mental health [3]. Normative sleep disturbances during adolescence include delayed sleep onset due to changes in circadian rhythms and changing sleep schedules due to school start times and extracurricular activities [4,5]. Understanding patterns of sleep disturbances during the adolescent developmental period is important because of their association with psychopathology, including internalizing and externalizing problems and post-traumatic stress symptoms [6,7]. Adolescents with higher levels of sleep disturbances, such as nightmares, fatigue, insomnia, and under- or oversleeping, tend to also report higher mental health symptom severity compared to those with lower levels of sleep disturbances [2,7]. Furthermore, approximately 50% of all mental disorders have their onset during adolescence [8], and sleep disturbances are also prevalent during this time, highlighting the critical need to examine their associations to inform potential targets for intervention. The current study used data from a nationally representative study of youth (we use the term “youth” to refer to both children and adolescents throughout the manuscript, unless we are referring to a specific study’s use of the term youth; we will use the term “adolescents” to refer to youth aged 11–17 years) involved with the U.S. child welfare system, a population exposed to high adversity, to identify patterns of sleep disturbances and their associations with internalizing, externalizing, and post-traumatic stress symptoms among adolescents (ages 11–17 years).

### 1.1. Sleep Disturbances and Internalizing Symptoms

Robust evidence links sleep disturbances (i.e., insomnia, fatigue, and difficulty initiating or maintaining sleep) with internalizing symptoms, as sleep disturbances are included in the diagnostic criteria for depression and anxiety. Prior studies have established that sleep disturbances are associated with higher levels of internalizing symptoms, including anxiety and depression, even if symptom levels do not meet clinical diagnostic standards [9,10]. Cross-sectional studies indicate that shorter sleep duration is associated with higher levels of depressive symptoms [11] and anxiety symptoms [12] in adolescents. Similar results have also been found in longitudinal studies. For example, under- and over-sleeping on weekdays and weekends have been linked to depressive symptoms in adolescents over a two-year longitudinal period [13], and shorter sleep duration predicted panic disorder and generalized anxiety disorder symptoms among adolescents [14]. In addition to examining specific disorders, research using composite scores of internalizing symptoms has found consistent associations with sleep disturbances. Using the Adolescent Brain Cognitive Development (ABCD) study of 11,670 early adolescents (9–10 years old) in the U.S., Goldstone and colleagues found that sleep disturbances associated with excessive somnolence (e.g., fatigue, sleeping more during the daytime) were associated with higher levels of internalizing symptoms at baseline and at a one-year follow-up [15].

### 1.2. Sleep Disturbances and Externalizing Symptoms

Existing evidence also suggests that sleep disturbances are associated with externalizing symptoms, including attention-deficit hyperactivity disorder (ADHD) and conduct disorder [16,17]. Among children with ADHD, multiple types of sleep disturbances have been self-reported, such as daytime fatigue, insomnia, and taking longer to fall asleep [18], and these findings have been supported by more objective assessments of sleep, including actigraphy [19,20] and polysomnography [21,22]. Prospectively, there is evidence that sleep disturbances pre-date ADHD diagnoses and that sleep disturbances during childhood predict ADHD symptoms in adolescence [23]. Sleep disturbances are also associated with conduct disorders. In a study by Fulfs and colleagues, total sleep disturbance scores as well as each individual type of sleep disturbance (e.g., sleep onset delay, night wakings, daytime fatigue, and shorter sleep duration) were associated with higher levels of conduct problems in a sample of children and adolescents [24]. Sleep onset delay, shorter sleep duration, and parasomnias had the strongest associations with conduct problems. Longitudinal associations between sleep disturbances and externalizing symptoms in the ABCD study suggest that sleep disturbances associated with initiating and maintaining sleep (e.g., sleeping less, night wakings) were significantly associated with higher levels of externalizing symptoms at baseline and at a one-year follow-up [15].

### 1.3. Sleep Disturbances and Post-Traumatic Stress Disorder (PTSD) Symptoms

Child welfare-involved adolescents are often exposed to disproportionate rates of childhood adversity, which may increase sleep disturbance associations with PTSD symptoms. However, much of the existing literature has examined sleep disturbances as a consequence of traumatic events, rather than considering the role of sleep disturbances in predicting PTSD symptoms. Some longitudinal evidence suggests a bidirectional association between sleep disturbances and PTSD symptoms, providing some evidence that sleep disturbances may predict PTSD symptoms. For example, Schneiderman et al. used longitudinal data from a study examining the effects of maltreatment on adolescent development and found that sleep disturbances assessed during the third timepoint significantly predicted PTSD symptoms approximately 4.5 years later at time 4 [25]. A systematic review of studies examining associations between sleep disturbances and PTSD symptoms provides further evidence that sleep disturbances (i.e., shorter sleep duration, poorer quality sleep) predict higher levels of PTSD symptoms [26]. The association between sleep disturbances, particularly insomnia, and PTSD symptoms may be due to an increase in hyperarousal following trauma that both disrupts sleep and worsens PTSD symptoms [9].

### 1.4. Patterns of Sleep Disturbances and Mental Health Symptoms

Despite the well-established relationship between sleep disturbances and mental health symptoms, relatively few studies have used person-centered approaches to identify qualitatively distinct subgroups of adolescents based on their patterns of sleep disturbances. Rather than assuming uniform effects, person-centered approaches, such as latent class analysis (LCA), allow researchers to identify distinct patterns of sleep disturbances based on responses to a set of indicators (e.g., types of sleep disturbances). This approach provides a more nuanced assessment of sleep disturbances by accounting for their co-occurrence and identifying heterogeneity in adolescent sleep by grouping individuals with similar patterns of sleep disturbance [27].

Using Fitbit sleep data from the ABCD study, latent profile analysis was conducted to identify subgroups of early adolescents based on average sleep duration, efficiency, latency (i.e., time to fall asleep), midpoint (i.e., midpoint between onset of sleep and waking), length of time awake during sleep period, and number of wakings during the sleep period [28]. This study identified four unique profiles characterized by (a) average sleep values, (b) shorter duration and low efficiency, (c) high efficiency, shorter duration and less wakefulness, and (d) longer duration and more wakefulness. The profiles characterized by shorter sleep duration tended to have higher externalizing problems (e.g., rule-breaking behaviors, attention problems) compared to profiles characterized by longer sleep durations and average sleep values; however, there were no differences in internalizing symptoms (e.g., anxious/depressed, withdrawn/depressed symptoms) across profiles. These results are similar to findings from the ABCD study that used broad classifications of sleep disturbances and found that disturbances associated with initiating and maintaining sleep (such as shorter sleep duration) were associated with higher levels of externalizing symptoms but not internalizing symptoms [15].

#### Sleep Disturbances and Child Welfare-Involved Youth

The potential heterogeneity in sleep disturbances is especially relevant for adolescents in child welfare contexts, who are disproportionately exposed to childhood adversity and at increased risk for sleep disturbances and mental health problems. Sleep disturbances are prevalent among child welfare-involved youth. Estimates suggest that as many as 54% of child welfare-involved youth experience some form of sleep disturbance [29,30,31]. The high rates of sleep disturbances in this population may be due to their high rates of adversity exposure and household dysfunction (e.g., irregular schedules, shared sleeping spaces, and nighttime stressors related to traumatic experiences), but they may also be due to the unique scenario of child welfare involvement, particularly out-of-home placement. For youth who are placed into out-of-home care, such as placement with a relative or a foster parent, changing sleep environments and routines, differences in shared sleep space arrangements, unfamiliarity with caregivers, and lack of emotional and physical security may contribute to sleep disturbances [32,33]. Yet, to date, person-centered approaches to sleep disturbances remain underutilized in child welfare samples, limiting insight into how sleep patterns cluster and relate to mental health risk in this high-risk population.

### 1.5. Current Study

The current study addresses limitations in the extant literature regarding the heterogeneity of sleep disturbances among child welfare-involved youth and the association between sleep disturbances and mental health symptoms over time. We used LCA to identify subgroups of adolescents involved with the child welfare system based on patterns of self-reported sleep disturbances. Then, we tested whether there were differences in internalizing, externalizing, and PTSD symptoms (at baseline and an 18-month follow-up) across subgroups of sleep disturbances, adjusting for the effects of covariates. We hypothesized that subgroups characterized by sleep disturbances related to initiating and maintaining sleep (e.g., sleeping less, trouble sleeping, and nightmares) would be associated with higher levels of externalizing and PTSD symptoms, and that subgroups characterized by sleep disturbances related to excessive somnolence (e.g., fatigue, sleeping more) would be associated with higher levels of internalizing symptoms compared to a subgroup characterized by no sleep disturbances.

## 2. Materials and Methods

### 2.1. Dataset and Sample

The data used in this study were collected as part of the second cohort of the National Survey on Child and Adolescent Wellbeing (NSCAW-II) [34]. NSCAW-II collected data from child welfare-involved youth, their caregivers, child welfare workers, and teachers between March 2008 and December 2012. Eligible youth received a child welfare investigation between February 2008 and April 2009. Youth were identified through a two-stage stratified sampling design, and sampling weights allow for a nationally representative sample of children in the U.S. who received investigations for alleged maltreatment and were located within jurisdictions that allowed NSCAW study staff to contact caregivers for informed consent (i.e., did not require child welfare agency staff to make first contact). The current study uses baseline and 18-month follow-up data. Baseline data were collected between March 2008 and September 2009, and follow-up data were collected between October 2009 and January 2011, approximately 18 months after baseline data collection. The current study used the provided sampling weights to obtain unbiased estimates and to achieve a nationally representative sample. IRB approval for use of the secondary dataset was provided by the first author’s university. Further information regarding NSCAW, its methodology, and sampling weights can be found in Biemer et al. [35] and Dolan et al. [36].

NSCAW-II included a sample of 5872 youth between 0 and 17.5 years at baseline. This included an oversampling of infants and children placed in out-of-home care, and an under-sampling of child welfare cases not receiving services. To focus on the adolescent developmental period, we narrowed our sample to adolescents aged 11–17 years at baseline (*N* = 1056, *M* age = 13.61 years, *SD* age = 1.86). The sample included slightly more females than males (59.5% and 40.5%, respectively). Most adolescents were living with biological or adoptive parents (*n* = 896.34, 84.9%), with only 15.1% of adolescents in out-of-home care at baseline (*n* = 159.66). Additional demographic information is provided in Table 1.

### 2.2. Measures

#### 2.2.1. LCA Indicators of Sleep Disturbances

We used five items from the Youth Self-Report (YSR) [37] as indicators of sleep disturbances. The items included the assessment of: (a) nightmares, (b) feeling overtired without a reason, (c) sleeping less than other kids, (d) sleeping more than other kids during the day and/or night, and (e) trouble sleeping. These items were distributed across multiple syndrome scales, including somatic complaints, thought problems, and other problems. Although the YSR is scored on a 3-point scale ranging from not true to often true, we collapsed the rating scale to not endorsed (=0, not true) and endorsed (=1, sometimes or often true) to create binary indicators for each of the sleep disturbance items. A sensitivity analysis using the 3-point ordinal scale is provided in the Supplemental Materials.

#### 2.2.2. Mental Health Symptoms

The YSR was also used for the assessment of internalizing and externalizing behavior problems [37]. The YSR includes a total of 112 items to assess emotional and behavioral problems in youth. Youth rank how true each item is of them on a 3-point scale ranging from not true to very/often true. Items are grouped into behavioral syndromes that can then be used to calculate internalizing (anxious/depressed, withdrawn/depressed, and somatic complaints) and externalizing scores (rule-breaking behavior, aggressive behavior). We used the normed *t*-scores for internalizing and externalizing behavior problems in the analysis. Scores between 65 and 69 are considered to be in the borderline clinically significant range, and scores 70 and greater are clinically significant. The internal consistency for internalizing (ω = 0.95 and 0.96) and externalizing behavior problems (ω = 0.95 and 0.96) at baseline and an 18-month follow-up, respectively, were good.

PTSD symptoms were assessed using the Trauma Symptom Checklist for Children post-traumatic stress subscale [38]. This subscale includes 10 items rated on a 4-point frequency scale ranging from never to almost all of the time. We used normed *t*-scores for the analysis. Scores 65 or greater are clinically significant; however, scores between 60 and 64 may be clinically relevant as they may indicate some difficulty due to post-traumatic stress symptomatology. The PTSD symptom items demonstrated good internal consistency at baseline and an 18-month follow-up (ω = 0.91 and 0.92).

#### 2.2.3. Covariates

We included a cumulative childhood adversity score and sociodemographic covariates to account for their influence on latent class membership and mental health symptoms. The childhood adversity items were obtained from multiple measures, including youth reports on the Parent-Child Conflict Tactics Scale (CTSPC) [39], parent reports on the Conflict Tactics Scale 2 (CTS2) [40], and study-specific questions and information provided by child welfare caseworkers regarding child welfare investigations and case history. We selected items from these measures that aligned with the 10 adverse childhood experiences (ACEs) [41]. Adolescents were considered exposed (=1) to the item if any reporter endorsed any of the items corresponding to the ACE and not exposed (=0) if there was no endorsement across reporter and items. For example, if the youth self-reported having experienced a form of physical abuse, but the caregiver did not endorse physical maltreatment of the child, the youth would be considered exposed to physical abuse (=1). A total sum score was calculated from the 10 binary items for use in analyses.

Sociodemographic variables were obtained based on youth and child welfare caseworker reports. Adolescents reported their age (in years), gender (1 = male, 2 = female), and race/ethnicity (0 = White/non-Hispanic, 1 = Black/non-Hispanic, Hispanic, Asian/Hawaiian/Pacific Islander, or American Indian). Child welfare caseworkers reported whether youth were in out-of-home care (=1) or living with biological or adoptive parents (=2).

### 2.3. Analytic Plan

All analyses were conducted using Mplus (version 8.10). The stratified sampling strategy NSCAW employed was accounted for in all analyses by using the complex data structure within Mplus (e.g., TYPE = COMPLEX MIXTURE) and accounting for the individual sampling weights (i.e., WEIGHT=) and probability sampling units (i.e., CLUSTER=) included in the NSCAW dataset. Missing data in the analyses were addressed using full information maximum likelihood (FIML). There were 15 adolescents missing on all sleep items. Therefore, the latent class analysis of sleep disturbances was conducted using a sample of 1041 youth. Due to missing on at least one covariate, subsequent analyses examining associations between class membership and mental health symptoms were conducted using a sample of 953 and 685 adolescents for analyses with internalizing and externalizing symptoms at baseline and the 18-month follow-up, respectively, and using a sample of 950 and 681 for analyses with PTSD symptoms at baseline and the follow-up, respectively.

Descriptive statistics and bivariate correlations were examined for all covariates, latent class indicators, and mental health symptom outcome variables (see Table 1 and Table 2). To identify subgroups of adolescents based on patterns of sleep disturbances, we conducted a series of latent class analyses (LCA) with each subsequent LCA including an additional latent class (see Table 3). The best-fitting model and number of subgroups was selected based on an evaluation of multiple model fit statistics and theoretical meaningfulness [42]. We evaluated fit based on the Bayesian information criterion (BIC), sample-size-adjusted BIC (SABIC), and approximate weight of evidence (AWE), with lower values indicating better model fit. We also considered the model entropy and odds of correct classification (OCC) as indicators of classification quality, such that entropy values closer to 1 are indicative of better quality and OCC values greater than 5 support adequate class separation and precision. The Lo-Mendell-Rubin (LMR-LRT) and Vuong-Lo-Mendell-Rubin likelihood ratio tests (VLMR-LRT) were examined to compare models. A significant LRT *p*-value provided evidence that the less parsimonious model (k + 1 model) was a better fit compared to a more parsimonious k model. We also considered the smallest class size, to ensure subgroups consisted of a meaningful number of adolescents.

After selecting the best-fitting model and optimal number of latent classes to capture the heterogeneity of sleep disturbances in our sample, we used the three-step Bolck-Croon-Hagenaars (BCH) approach to examine subgroup differences in covariates and mental health outcomes [43]. The BCH approach allows predictors and distal outcomes to be included in the same model while also stabilizing latent class membership in these analyses and accounting for classification error at the individual level through the use of weights [44]. Using the BCH approach and weights, we examined whether there were mean differences in mental health scores based on latent class membership, adjusting for the effects of the covariates. Each mental health score was regressed on the latent class variable and the predictors. Separate Wald tests were conducted to determine whether there was a significant mean difference in mental health scores. A Bonferroni correction was applied to our interpretation of *p*-values to prevent an inflated Type I error rate due to examining differences across multiple mental health scores at Time 1 and Time 2. Based on conducting 6 separate Wald tests, our Bonferroni-adjusted significance level suggested that *p*-values for the Wald tests would need to be less than 0.0083 to indicate a significant mean difference in mental health scores. Significant mean differences based on latent class membership were further examined by calculating mean differences for each latent class pair (e.g., class 1 versus class 2) using the Mplus model constraint function. Standardized effects were calculated to provide effect sizes of the mean differences.

## 3. Results

### 3.1. Descriptive Statistics

Approximately half of the sample endorsed experiencing each sleep disturbance at least some of the time. The most frequently endorsed sleep disturbance was “nightmares” (50.8%), followed by “sleeping more than most kids during the day and night” (47.7%), “trouble sleeping” (46.4%), and “feeling overtired without good reason” (45.9%). The least frequently endorsed sleep disturbance was “sleeping less than most kids” (42.8%). The weighted frequencies of endorsing each sleep disturbance item are provided in Table 1 and weighted correlations are shown in Table 2. Regarding correlations among the mental health dependent variables, post-traumatic stress symptoms, internalizing behavior problems, and externalizing behavior problems at baseline and an 18-month follow-up were all moderately and positively correlated (*r*s range: 0.26–0.68). All the sleep disturbance variables, except for sleeping less than most kids, were also positively correlated with the mental health variables (*r*s range: 0.19–0.56).

### 3.2. Latent Class Enumeration

The fit indices of the latent class analysis are provided in Table 3. Examining the fit statistics across models, the BIC, SABIC, and AWE supported the three-class model. The BIC and SABIC continued to decrease from the one-class model to the three-class model, which had the lowest values. The AWE also supported the three-class model. As a conservative fit index, the lowest AWE value in the two-class model provides support for the three-class model. However, the LRTs indicated that the additional model complexity of the three-class model was not a significant improvement compared to the two-class model, which was also supported by a higher entropy value. We examined both the two-class and three-class models in more detail based on the theoretical meaningfulness of the identified subgroups. The two-class model identified two subgroups characterized by either low probability of endorsing any sleep disturbance or high probability of endorsing multiple sleep disturbances. The two-class model did not provide theoretically meaningful subgroups beyond what could be assessed using a sum score of endorsed sleep disturbances. In contrast, the three-class model identified three distinct subgroups characterized by different patterns of endorsed sleep disturbances; therefore, this three-class solution was retained for subsequent analyses. The three-class model demonstrated adequate class separation and within-class homogeneity, as indicated by the OCC and average posterior class probabilities (range: 0.71–0.91). Further comparisons of the two- and three-class models are provided in Supplemental Table S5. Additionally, a sensitivity analysis of the enumeration process using the ordinal response scale (not true, sometimes true, very true) is provided in Supplemental Tables S1–S5 and Figures S1–S3.

**Figure 1:**
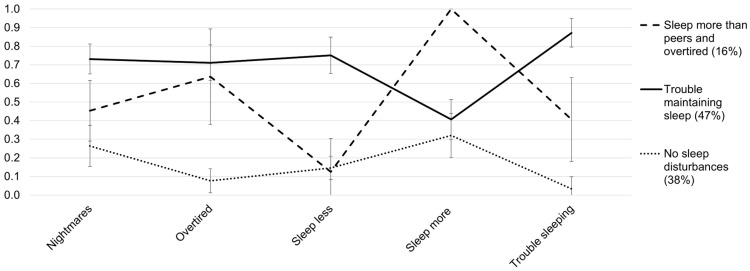
Item probability plot for the three-class model (*N* = 1041). The error bars represent 95% confidence intervals.

We named the three-class solution according to the patterns in sleep disturbance item endorsement (see Figure 1 and Supplemental Table S6). Class 1 included 16% of the sample and was labeled the *sleeping more than peers and overtired* subgroup due to the high probability of sleeping more (>0.70) and feeling overtired (0.64). Although the feeling overtired probability was less than 0.70 in this subgroup, it was close to the cut-off and much higher in comparison to the *no sleep disturbance* subgroup (0.08). The second class (47% of the sample) was characterized by high probabilities (>0.70) of endorsing nightmares, feeling tired, sleeping less than peers, and having trouble sleeping. Thus, this class was labeled the *trouble maintaining sleep* subgroup. Class 3 (38% of sample) was labeled the *no sleep disturbances* subgroup as it was characterized by a low probability (<0.30) of endorsing all sleep disturbance items.

### 3.3. Latent Class Differences in Mental Health

**Figure 2:**
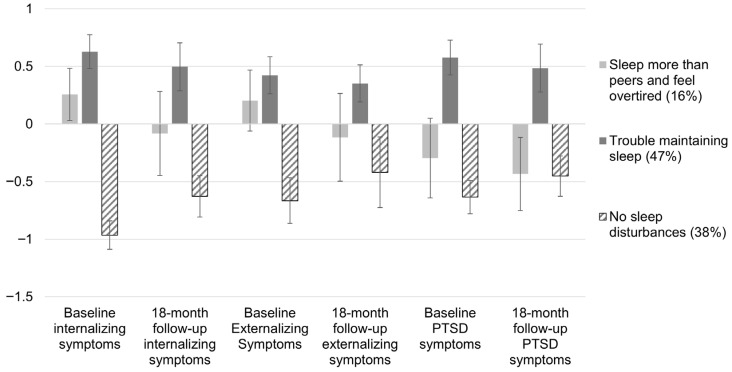
Mean scores of internalizing, externalizing, and PTSD symptoms at baseline and an 18-month follow-up across subgroups. *N* = 1041. *Y*-axis values represent standard deviations from the sample mean. Error bars represent 95% confidence intervals.

The standardized means for internalizing, externalizing, and PTSD symptoms for each of the latent classes are provided in Figure 2. At baseline, there were significant differences across subgroups for internalizing behavior problems, X^2^(2) = 216.72, *p* < 0.001. Adolescents in the *sleeping more than peers and overtired* subgroup (adjusted *M* = 58.21, 95% CI: 34.05, 82.79; *d* = 1.27, *p* < 0.001, 95% CI: 0.91, 1.63) and the *trouble maintaining sleep* subgroup (adjusted *M* = 62.19, 95% CI: 38.34, 86.04; *d* = 1.58, *p* < 0.001, 95% CI: 1.40, 1.76) reported greater internalizing symptoms compared to the *no sleep disturbances* subgroup (adjusted *M* = 44.13, 95% CI: 19.32, 68.94). There was not a significant difference in internalizing behavior problems between the *sleeping more than peers and overtired* and *trouble maintaining sleep* subgroups (*d* = −0.31, *p* = 0.058, 95% CI: −0.63, 0.01). When examining whether there were subgroup differences in internalizing behavior problems measured at an 18-month follow-up, only one pairwise difference remained significant, X^2^(2) = 72.66, *p* < 0.001. The *trouble maintaining sleep* subgroup (adjusted *M* = 54.82, 95% CI: 49.10, 60.53) had greater internalizing behavior problems compared to the *no sleep disturbances* subgroup (adjusted *M* = 42.39, 95% CI: 36.83, 47.92; *d* = 1.08, *p* < 0.001, 95% CI: 0.82, 1.35). There were no significant differences in internalizing behavior problems at an 18-month follow-up between the *sleeping more than peers and overtired* subgroup (adjusted *M* = 48.59, 95% CI: 39.50, 57.68) and either the *trouble maintaining sleep* subgroup (*d* = −0.55, *p* = 0.089, 95% CI: −1.18, 0.08) or the *no sleep disturbances* subgroup (*d* = 0.54, *p* = 0.065, 95% CI: −0.03, 1.10).

Externalizing behavior problems similarly differed across subgroups at baseline, X^2^(2) = 63.51, *p* < 0.001. Adolescents in the *sleeping more than peers and overtired* subgroup (adjusted *M* = 59.64, 95% CI: 51.01, 68.26) and the *trouble maintaining sleep* subgroup (adjusted *M* = 63.04, 95% CI: 56.06, 70.02) had higher externalizing behavior problems compared to the *no sleep disturbances* subgroup (adjusted *M* = 44.13, 95% CI: 19.32, 68.94; *d* = 0.79, *p* < 0.001, 95% CI: 0.44, 1.15; and *d* = 1.07, *p* < 0.001, 95% CI: 0.81, 1.34, respectively). Although the *sleeping more than peers and overtired* subgroup had lower externalizing behavior problems scores than the *trouble maintaining sleep* subgroup, the difference was not statistically significant (*d* = −0.28, *p* = 0.084, 95% CI: −0.60, 0.04). Externalizing behavior problems at an 18-month follow-up significantly differed across subgroups, X^2^(2) = 25.49, *p* < 0.001. At the follow-up, the *sleeping more than peers and overtired* subgroup (adjusted *M* = 51.27, 95% CI: 41.60, 60.93) had significantly lower externalizing behavior problems than the *trouble maintaining sleep* subgroup (adjusted *M* = 58.71, 95% CI: 51.39, 66.03; *d* = −0.60, *p* = 0.008, 95% CI: −1.05, −0.16). The *trouble maintaining sleep* subgroup continued to have higher externalizing behavior problems compared to the *no sleep disturbances* subgroup (adjusted *M* = 49.35, 95% CI: 43.20, 55.51; *d* = 0.75, *p* < 0.001, 95% CI: 0.41, 1.08).

All subgroups significantly differed in their PTSD symptoms at baseline, X^2^(2) = 121.67, *p* < 0.001, such that subgroups characterized by worse sleep disturbances had higher PTSD symptoms. Specifically, the *trouble maintaining sleep* subgroup (adjusted *M* = 61.52, 95% CI: 57.75, 65.29) had higher scores than the *sleeping more than peers and overtired* subgroup (adjusted *M* = 53.42, 95% CI: 47.79, 59.05; *d* = 0.77, *p* = 0.001, 95% CI: −1.20, −0.33) and the *no sleep disturbances* subgroup (adjusted *M* = 48.35, 95% CI: 44.49, 52.21; *d* = 1.22, *p* < 0.001, 95% CI: 1.01, 1.44). Adolescents in the *sleeping more than peers and overtired* subgroup also had higher PTSD symptoms than the *no sleep disturbances* subgroup (*d* = 0.46, *p* = 0.020, 95% CI: 0.07, 0.85). Most of the significant differences in PTSD symptoms across subgroups were retained at an 18-month follow-up, X^2^(2) = 51.13, *p* < 0.001. The *trouble maintaining sleep* subgroup (adjusted *M* = 57.09, 95% CI: 50.07, 64.10) continued to have higher PTSD symptoms at the follow-up compared to the *sleeping more than peers and overtired* subgroup (adjusted *M* = 48.47, 95% CI: 40.64, 56.31; *d* = 0.93, *p* < 0.001, 95% CI: −1.38, −0.48) and the *no sleep disturbances* subgroup (adjusted *M* = 48.30, 95% CI: 41.68, 54.92; *d* = 0.93, *p* < 0.001, 95% CI: 0.66, 1.20). Unlike at baseline, the difference in PTSD symptoms at the follow-up between the *sleeping more than peers and overtired* subgroup and the *no sleep disturbances* subgroup was not significant (*d* = −0.004, *p* = 0.986, 95% CI: −0.46, 0.45).

## 4. Discussion

The goals of this study were to (a) identify underlying subgroups of adolescents based on patterns of sleep disturbances and (b) examine whether internalizing, externalizing, and PTSD symptoms differ across subgroups. With regard to the first aim, we identified three latent subgroups based on patterns of sleep disturbance endorsement. The majority of adolescents were in the *trouble maintaining sleep* subgroup (47%), followed by the *no sleep disturbances* subgroup (38%), and 16% of youth were in the *sleeping more than peers and overtired* subgroup. These subgroups generally align with existing studies that identified latent subgroups of sleep among adolescents. For example, in general population samples of adolescents, prior studies have identified subgroups characterized by normative sleep or no sleep disturbances, at least one subgroup characterized by multiple types of sleep disturbance, and various other subgroups characterized by a single type of disturbance, such as night awakenings [28,45,46]. An additional dissertation study that included a sample of children (aged 3–11 years) adopted from foster care found similar subgroups of sleep—a normative sleep subgroup, subgroups characterized by a single type of sleep disturbance (e.g., sleep onset problems), and subgroups characterized by multiple types of sleep disturbances [47]. Due to differences in sleep indicators and samples, the exact characterizations of subgroups based on sleep disturbances differed across existing studies and the current study. Future studies should continue to examine subgroups of sleep disturbances to confirm the existence of these subgroups within child welfare populations.

Our second aim was to examine whether there were differences in mental health symptoms based on subgroup membership. Our findings suggest that those in the *no sleep disturbance* subgroup had the lowest levels of internalizing, externalizing, and PTSD symptoms at baseline and an 18-month follow-up, which was anticipated. Our hypotheses that a subgroup characterized by sleep disturbances related to initiating and maintaining sleep would be associated with higher levels of externalizing and PTSD symptoms compared to a subgroup characterized by no sleep disturbances based on Goldstone et al. [15] were partially supported. The *trouble maintaining sleep* subgroup endorsed items associated with issues initiating and maintaining sleep, including nightmares, sleeping less than peers, trouble sleeping, and feeling overtired. However, our findings suggested that both the *trouble maintaining sleep* and the *sleeping more than peers and overtired* subgroups reported higher levels of internalizing, externalizing, and PTSD symptoms at baseline compared to the *no sleep disturbances* subgroup. Those in the *trouble maintaining sleep* subgroup continued to have significantly higher levels of symptoms across all three symptom domains at the 18-month follow-up compared to the *no sleep disturbances* subgroup. One reason for the continuation of higher levels of symptoms over 18 months for the *trouble maintaining sleep* subgroup may be that multiple sleep disturbances resulting in less sleep influence neurobiological processes (e.g., hyperarousal, emotion regulation, and stress response) underlying mental health symptoms, leading to the persistence of mental health symptoms over time [48,49]. For example, within the context of child welfare-involvement, youth who have difficulty sleeping may experience disrupted stress response or emotion regulation that is compounded by exposure to childhood adversity, which, in turn, increases risk for mental health symptoms. There is also evidence that sleep deprivation (such as from issues with initiating and maintaining sleep), particularly during adolescence, may also influence structural and functional cognitive development [3]. For example, the prefrontal cortex is one structure that undergoes significant maturation processes during adolescence that increases vulnerability to sleep deprivation during this developmental period. Future research should prospectively examine how sleep disturbances impact mental health via underlying neurobiological processes to better understand the causal mechanisms and how to best intervene to promote improved sleep and mental health outcomes.

We anticipated that those in a subgroup characterized by excessive somnolence (i.e., the *sleeping more than peers and overtired* subgroup) would have higher levels of internalizing symptoms compared to those in the *no sleep disturbance* subgroup. Although that hypothesis was true at baseline, there were no longer significantly different levels of symptoms at the 18-month follow-up. One reason for this change at the follow-up may be that there was a decrease in all three symptoms between baseline and the follow-up in the *sleeping more than peers and overtired* subgroup (see Figure 2). Although our study cannot provide causal claims regarding the relationship between sleep and lower levels of symptoms at the 18-month follow-up, there is evidence that a longer sleep duration (i.e., sleeping more) is associated with lower levels of poor mental health symptoms over time [50]. Within our population of youth involved with the child welfare system, longer sleep duration may serve as a protective factor over time, buffering against the effects of exposure to childhood adversity on mental health symptoms. Sleeping more may provide cognitive structural and functional benefits via similar pathways as sleep deprivation, such as through the prefrontal cortex and other systems that promote regulation of emotion processing and the stress response system, known factors associated with mental health [51,52,53]. It is important to continue to disentangle the benefits of longer sleep durations that meet sleep recommendations and the risk of sleeping longer without feeling rested (i.e., hypersomnia) for depression and other mental disorders.

We did not make specific hypotheses regarding differences in mental health symptoms between subgroups characterized by trouble initiating and maintaining sleep and by excessive somnolence. Our results indicated that there were no significant differences in internalizing symptoms between the two subgroups at baseline or an 18-month follow-up. However, the *trouble maintaining sleep* subgroup had significantly higher levels of externalizing symptoms compared to the *sleeping more than peers and overtired* subgroup at the 18-month follow-up. These findings are similar to those of Zhang et al. [28], who found that a subgroup characterized by low sleep duration and efficiency had the highest levels of externalizing symptoms (attention problems, rule-breaking behaviors, and social problems). Additionally, the *sleep more than peers and overtired* subgroup had lower levels of PTSD symptoms in comparison to the *trouble maintaining sleep* subgroup at baseline and the follow-up. These results also provide support that longer sleep duration may serve as a protective factor for externalizing and PTSD symptoms. Future research should explore these differences further and explore whether longer sleep duration is protective by using prospective, longitudinal data.

### Limitations

The findings of our study should be considered in light of the limitations. Although we used multiple reporters for the assessment of childhood adversity, we relied on adolescents’ self-report of sleep disturbance items and their mental health symptoms. As with the assessment of childhood adversity, there may be sleep disturbances that occur during the night that youth are unaware of that may influence their sleep. Further, the assessment of sleep disturbances was limited to the five sleep-related items in the YSR, which is not a sleep-specific measure, and item responses were dichotomized into endorsed versus not endorsed. Future research should use sleep-specific self-report scales and objective assessments of sleep, such as actigraphy, to gain a more comprehensive measurement of youth sleep disturbances that can provide more information regarding the frequency and severity of sleep disturbances. The LCA was also an exploratory, sample-specific analysis. Replication to confirm the existence of the identified subgroups is needed with sleep-specific measures that can be compared to existing sleep phenotypes. Until confirmation of our identified sleep disturbance subgroups, it is important to not overlook the potential unique effects of specific sleep disturbance types on mental health symptoms. Using items from the YSR to assess sleep and using the YSR to assess internalizing and externalizing symptoms may also have artificially inflated our estimates of the associations between sleep and symptom scores. However, only two sleep items were included in the scoring of internalizing symptoms (6.5% of 31 items). Our assessment of childhood adversity was also limited to the items included in the NSCAW study, which were aligned with the original 10 adverse childhood experiences. There has been recent research focused on expanding the assessment of adversity to include additional types of adversity, such as bullying victimization, community violence, placement into foster care, and identity-based discrimination and victimization [54,55,56]. An additional limitation is the two-time-point design that prevents us from establishing any causal connection between sleep disturbances and mental health symptoms. Future research should examine whether changes in mental health symptoms over time can be predicted by patterns of sleep disturbances. Additionally, prospective, longitudinal research examining relationships among all of the constructs over time is necessary to better understand associations between sleep and mental health symptoms, and how other neurobiological factors, such as hyperarousal, influence those associations in adolescence. Research that includes neuroimaging to assess neurobiological structural factors implicated in sleep and mental health symptoms is also needed within the high-risk child welfare population [57,58,59]. Future research should also include additional covariates that may influence sleep that were not accounted for in the current study, such as physical activity or the use of medications (e.g., stimulants), and should include an examination of interaction effects (e.g., sex, placement type, and medication use).

## 5. Conclusions

Our results highlight the importance of reducing sleep disturbances and promoting healthy sleep duration that meets the recommendations for adolescents to address mental health problems among child welfare-involved adolescents. Given the role of sleep in optimal youth health and development, there have been recent recommendations to focus on improving sleep for youth involved with the child welfare system [32,60]. There is a need for information and training for child welfare workers and all caregivers of youth (biological, kinship, foster, and adoptive) on trauma-informed approaches to improving sleep. Further research is necessary to inform these efforts. Existing research on sleep among children and adolescents involved with the child welfare system and more broadly in the context of exposure to adversity has often relied on total scores of sleep disturbances or focused on a single type of disturbance. This overlooks the potential heterogeneity in the types of sleep disturbances these youth experience, which may obscure differences in patterns of sleep disturbances that may be uniquely associated with psychopathology. For example, although the current study identified subgroups characterized by no sleep disturbances and multiple sleep disturbances (i.e., the *trouble maintaining sleep* subgroup) which may have been identified by a total score of sleep disturbances, the identification of the *sleep more than peers and overtired* subgroup may have been masked by a total score. Finally, our findings suggest that future research should continue to focus on identifying sleep disturbance patterns that increase risk for mental health symptoms, which has implications for interventions targeted at improving sleep.

## Figures and Tables

**Table 1:** Descriptive statistics (*N* = 1041).

Variables	Weighted		Unweighted	
	*M*/#	*SD*/%	*M*/#	*SD*/%
Age (in years)	13.63	1.86	13.73	1.86
Gender				
Male	420.56	40.4%	461	44.3%
Female	620.44	59.6%	580	55.7%
Out-of-Home Placement				
Yes	156.39	15.0%	337	32.4%
No	884.61	85.0%	704	67.6%
Race/Ethnicity				
White/non-Hispanic	447.64	44.5%	397	39.5%
Black/non-Hispanic	204.14	20.3%	280	27.9%
American Indian	123.80	12.3%	124	12.3%
Hispanic/multi-racial	229.28	22.8%	204	20.3%
Total adverse childhood experiences score	4.17	1.87	4.12	1.79
Mental Health Symptoms				
PTSD t-score at baseline	49.51	10.61	49.81	10.19
PTSD t-score at an 18-month follow-up	47.38	9.48	48.00	9.23
Internalizing t-score at baseline	49.72	11.50	49.57	11.34
Internalizing t-score at an 18-month follow-up	48.15	11.37	47.77	11.06
Externalizing t-score at baseline	54.32	12.05	53.81	12.26
Externalizing t-score at an 18-month follow-up	52.77	12.48	52.96	11.71
Sleep Disturbances (=endorsed)				
Nightmares	538.18	51.7%	527	50.8%
Fatigue	492.45	47.4%	476	45.9%
Sleeping less than most kids	444.61	42.8%	443	42.8%
Sleeping more than most kids	497.47	47.8%	494	47.7%
Trouble sleeping	514.17	49.5%	482	46.4%

**Table 2:** Summary of weighted correlations for key study variables (*N* = 1041).

	1	2	3	4	5	7	8	9	10	11	12	13	14	15	16	17
1. Age	-															
2. Gender	0.02	-														
3. Race/ethnicity	−0.05	0.02	-													
4. OOH placement	−0.17	0.10	−0.06	-												
5. Childhood adversity	−0.06	0.10	0.16	−0.11	-											
7. PTS symptoms T1	0.01	−0.09	0.09	−0.08	0.14	-										
8. PTS symptoms T2	0.12	−0.06	0.06	−0.10	0.05	0.54	-									
9. Internalizing T1	0.03	0.05	0.03	−0.03	0.14	0.68	0.4	-								
10. Internalizing T2	0.02	0.08	0.12	−0.01	0.08	0.47	0.70	0.59	-							
11. Externalizing T1	0.13	0.16	0.01	−0.04	0.14	0.41	0.28	0.59	0.45	-						
12. Externalizing T2	0.06	0.17	−0.01	−0.04	0.10	0.26	0.51	0.35	0.63	0.67	-					
13. Nightmares	−0.08	0.11	0.04	0.07	0.14	0.43	0.23	0.37	0.27	0.24	0.19	-				
14. Feeling overtired	−0.01	0.09	0.08	0.04	0.06	0.32	0.23	0.56	0.4	0.38	0.26	0.24	-			
15. Sleep less	−0.05	−0.04	0.04	0.02	0.08	0.35	0.31	0.40	0.33	0.30	0.26	0.22	0.31	-		
16. Sleep more	0.10	−0.05	−0.03	0.10	0.02	0.01	−0.01	0.10	0.01	0.12	0.03	−0.03	0.12	−0.11	-	
17. Trouble sleeping	−0.01	0.07	−0.04	0.05	0.06	0.41	0.31	0.52	0.36	0.34	0.26	0.35	0.41	0.44	0.02	-

Note. Gender = female; race/ethnicity = minoritized racial/ethnic group; and OOH placement not living with biological or adoptive parent.

**Table 3:** Fit indices for unconditional latent class models with one–six classes.

*k*	Par	LL	AIC	BIC	SABIC	AWE	VLMR-LRT *p*-Value	LMR-LRT *p*-Value	Entropy	Condition #	Smallest *n*
1	5	−3588.27	7186.54	7211.28	7195.40	7261.02				9.79 × 10^−1^	1041
2	11	−3313.15	6648.31	6702.73	6667.80	6812.16	0.024	0.026	0.73	7.66 × 10^−3^	468
**3**	**17**	**−3278.49**	**6590.99**	**6675.10**	**6621.11**	**6844.22**	**0.104**	**0.109**	**0.70**	**4.51 × 10^−3^**	**162**
4	23	−3271.00	6587.99	6701.80	6628.75	6930.60	0.605	0.608	0.81	5.17 × 10^−3^	96
5	29	−3265.61	6589.22	6732.71	6640.60	7021.20	0.535	0.537	0.84	9.29 × 10^−7^	77
6		Model did not converge.									

Note. *N* = 1041; *k* = number of classes; Par = number of parameters; LL = log likelihood; AIC = Akaike information criterion; BIC = Bayesian information criterion; SABIC = sample-size-adjusted BIC; AWE = approximate weight of evidence; VLMR-LRT = Vuong-Lu-Mendell-Rubin likelihood ratio test; and LMR-LRT = Lu-Mendell-Rubin likelihood ratio test. The bold values indicate the selected model.

## Data Availability

The NSCAW dataset used in the current study can be obtained through the National Data Archive on Child Abuse and Neglect: https://www.ndacan.acf.hhs.gov/index.cfm (accessed on 2 March 2026).

## Supplementary Materials

The following supporting information can be downloaded at: https://www.mdpi.com/article/10.3390/children13040441/s1, Table S1: Fit indices for unconditional latent class models with 1-6 classes using ordinal response scale; Table S2: Item response probabilities for the 2-class model using ordinal response scale; Figure S1: Item probability plot for the 2-class model (*N* = 1041); Table S3: Item response probabilities for the 3-class model using ordinal response scale; Figure S2: Item probability plot for the 3-class model (*N* = 1041); Table S4: Item response probabilities for the 4-class model using ordinal response scale; Figure S3: Item probability plot for the 4-class model (*N* = 1041); Table S5: Classification quality of the enumerated 2-class and 3-class models with binary response scale; Table S6: Item response probabilities for the 3-class model using binary response scale.
