# Supplementary material for: Associations Between Patterns of Sleep Disturbances and Mental Health Among Child Welfare-Involved Adolescents
Source: Children (Basel). 2026 Mar 24;13(4):441. doi: 10.3390/children13040441 (PMC13114552; doi:10.3390/children13040441)
Supplement: Supplementary file 1 [file children-13-00441-s001.zip › children-3995914-supplementary.pdf]

Supplementary Materials

Supplemental Table S1. Fit indices for unconditional latent class models with 1-6 classes using ordinal response scale.

k	Par	LL	AIC	BIC	SABIC	AWE	VLMR-LRT p-value	LMR-LRT p-value	Entropy	BF (k, k+1)	Condition #	Smallest *n*
1	10	662.24	10161.36	10210.84	10179.08	10310.31	-	-	1.00	7.56E-126	0.248E+00	1041
2	21	411.77	9530.72	9634.63	9567.93	9843.54	.004	.005	.709	1.74E-41	0.117E-02	457
**3**	**32**	291.67	9288.59	**9446.93**	9345.289	**9765.26**	**0.423**	**0.429**	**.750**	6.23E-01	0.987E-04	181
4	43	253.62	9233.22	9445.98	**9309.404**	9873.74	0.478	0.481	.774	**7.29E+05**	0.482E-04	177
5	54	227.82	9205.79	9472.98	9301.47	10010.17	Did not converge.		.809	**7.08E+06**	7.08E+06	116
6	65	206.49	9182.91	9504.52	9298.07	10151.14	0.770	0.770	.828	-	0.782E-09	69

Note. N = 1,041; *k* = number of classes, Par = number of parameters, LL = log likelihood, AIC = Akaike information criterion, BIC = Bayesian information criterion, SABIC = sample-size adjusted BIC, AWE = approximate weight of evidence, VLMR-LRT = Vuong-Lu-Mendell-Rubin likelihood ratio test, LMR-LRT = Lu-Mendell-Rubin likelihood ratio test, BF = Bayes Factor comparing k-class and k+1 class. The bolded values indicate the values that provide support for identifying the best-fitting latent class model.

The fit indices for the models using the ordinal response scale provide support for the 2-class model (LRTs), 3-class model (BIC), and 4-class model (SABIC, AWE, BF), respectively. We explored item response probabilities for each of these models below.

Supplemental Table S2. Item response probabilities for the 2-class model using ordinal response scale.

Sleep item	Response option	Latent Subgroups		Class Separation
		Class 1	Class 2	
**2-class model**		**43.92%**	**57.08%**	**1 vs. 2**
**Nightmares**	Not true	0.26	0.67	0.17
	Sometimes true	0.50	0.33	2.04
	Very true	0.24	0.00	161.05
**Feel overtired without good reason**	Not true	0.24	0.76	0.10
	Sometimes true	0.52	0.22	3.74
	Very true	0.24	0.02	18.06
**Sleep less than most kids**	Not true	0.25	0.84	0.06
	Sometimes true	0.47	0.13	6.19
	Very true	0.28	0.03	11.40
**Sleep more than most kids**	Not true	0.54	0.51	1.12
	Sometimes true	0.32	0.31	1.02
	Very true	0.15	0.18	0.79
**Trouble sleeping**	Not true	0.12	0.82	0.03
	Sometimes true	0.53	0.18	5.09
	Very true	0.35	0.00	5479.33

Note. N = 1,041. Cells are highlighted in blue to indicate probabilities that are generally different than chance (i.e., probability > 0.70 or < 0.30) or highlighted in green to indicate adequate class separation (i.e., >5 or < 0.2).

Figure S1. Item probability plot for the 2-class model (N = 1,041).

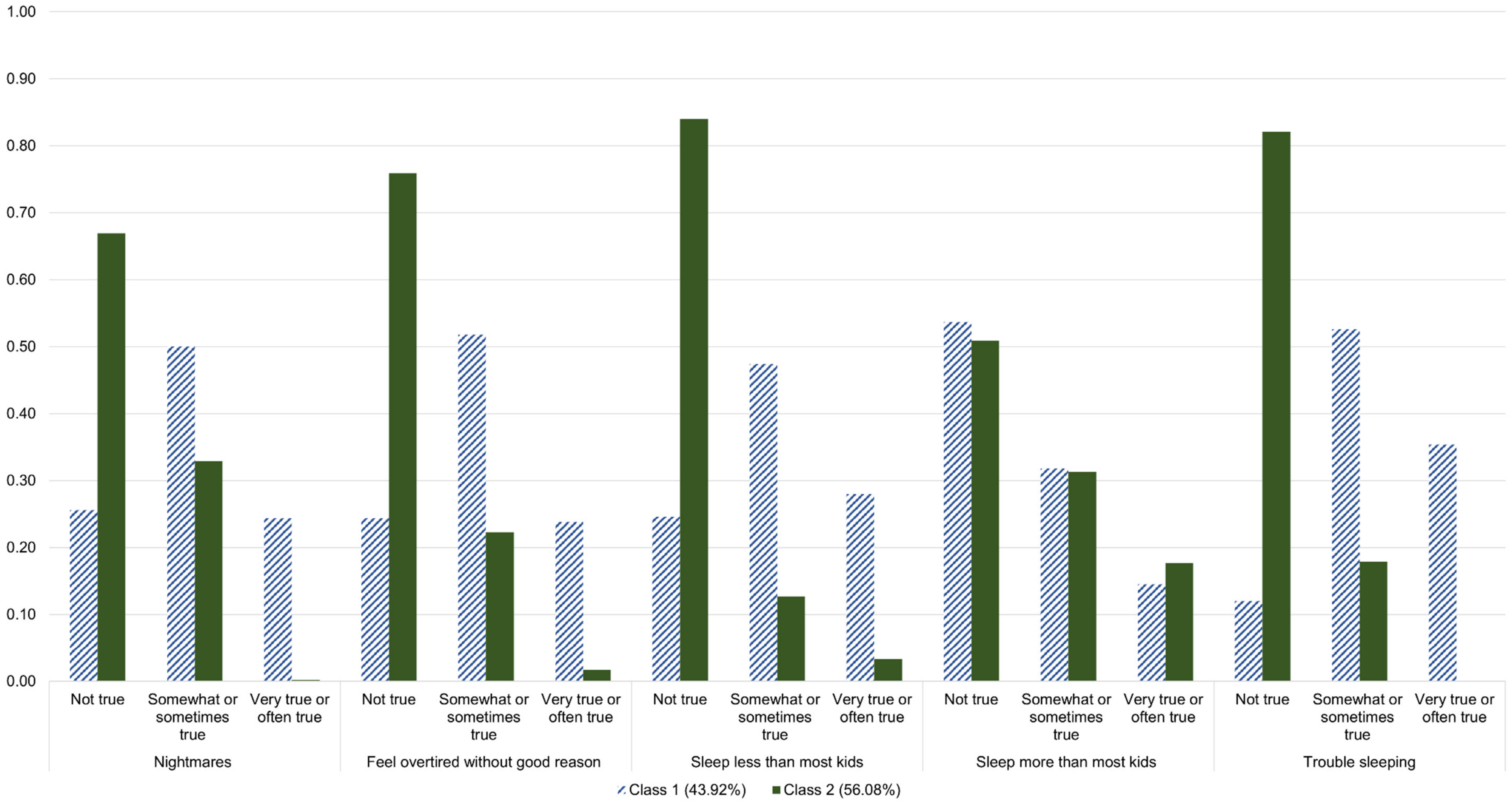

Supplemental Table S3. Item response probabilities for the 3-class model using ordinal response scale.

Sleep item	Response option	Latent Subgroups			Class Separation		
		Class 1	Class 2	Class 3			
**3-class model**		**37.27%**	**17.38%**	**45.35%**	**1 vs. 2**	**1 vs. 3**	**2 vs. 3**
**Nightmares**	Not true	0.33	0.21	0.72	1.81	0.19	0.10
	Sometimes true	0.57	0.38	0.28	2.18	3.43	1.57
	Very true	0.10	0.41	0.00	0.16	113.47	694.22
**Feel overtired without good reason**	Not true	0.34	0.19	0.82	2.13	0.11	0.05
	Sometimes true	0.61	0.29	0.17	3.97	7.73	1.95
	Very true	0.05	0.52	0.01	0.05	4.00	82.91
**Sleep less than most kids**	Not true	0.35	0.28	0.87	1.41	0.08	0.06
	Sometimes true	0.62	0.06	0.09	23.46	15.83	0.67
	Very true	0.03	0.66	0.04	0.02	0.78	48.28
**Sleep more than most kids**	Not true	0.42	0.65	0.55	0.39	0.60	1.54
	Sometimes true	0.50	0.07	0.26	12.99	2.93	0.23
	Very true	0.07	0.27	0.19	0.21	0.33	1.58
**Trouble sleeping**	Not true	0.14	0.18	0.94	0.78	0.01	0.02
	Sometimes true	0.70	0.24	0.07	7.30	33.89	4.64
	Very true	0.15	0.58	0.00	0.13	1806.19	13695.31

Note. N = 1,041. Cells are highlighted in blue to indicate probabilities that are generally different than chance (i.e., probability > 0.70 or < 0.30) or highlighted in green to indicate adequate class separation (i.e., >5 or < 0.2).

Figure S2. Item probability plot for the 3-class model (N = 1,041).

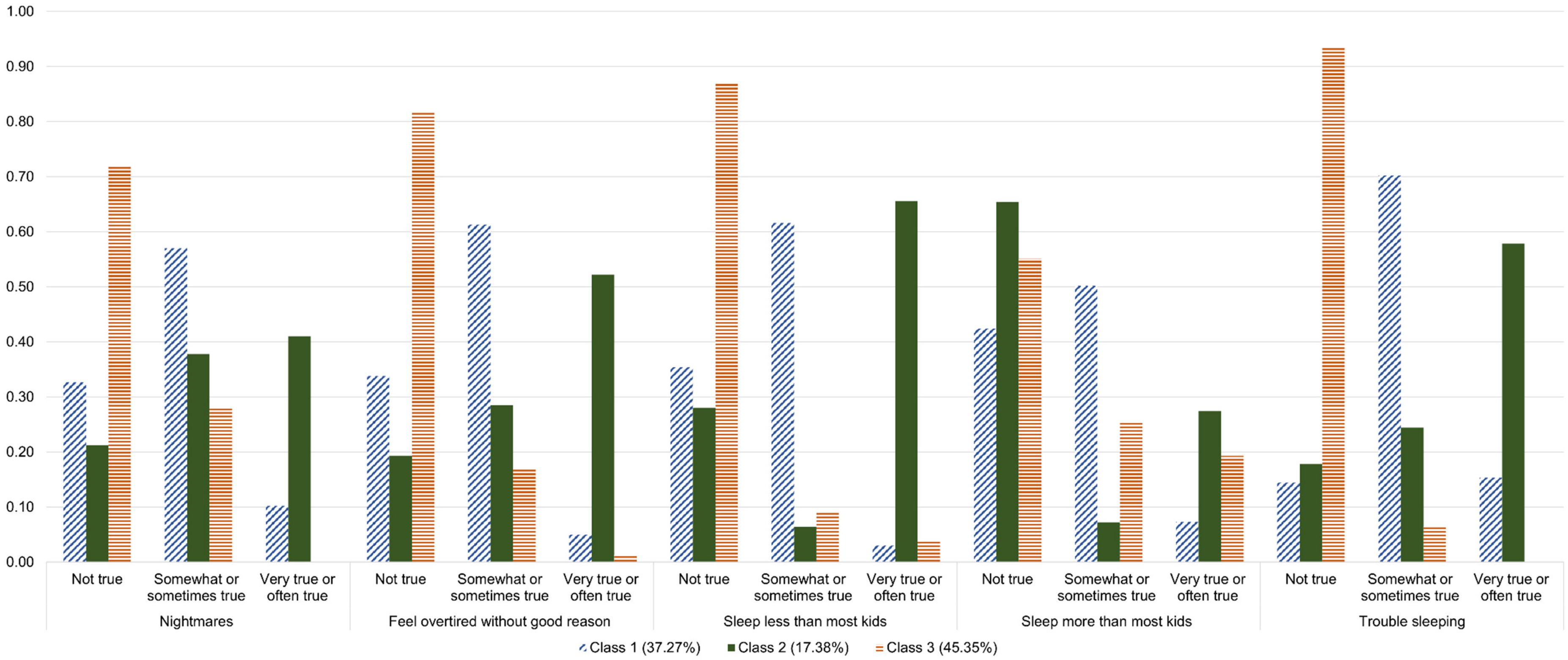

Supplemental Table S4. Item response probabilities for the 4-class model using ordinal response scale.

Sleep item	Response option	Latent Subgroups				Class Separation					
		Class 1	Class 2	Class 3	Class 4						
**4-class model**		**25.20%**	**23.68%**	**34.16%**	**16.96%**	**1 vs. 2**	**1 vs. 3**	**1 vs. 4**	**2 vs. 3**	**2 vs. 4**	**3 vs. 4**
**Nightmares**	Not true	0.33	0.50	0.77	0.21	0.50	0.15	0.30	1.79	3.61	12.02
	Sometimes true	0.54	0.50	0.23	0.36	1.16	3.92	3.38	2.08	1.79	0.53
	Very true	0.13	0.00	0.00	0.42	1494.10	49.66	0.00	0.20	0.00	0.00
**Feel overtired without good reason**	Not true	0.32	0.45	0.97	0.20	0.58	0.01	0.02	1.83	3.18	132.27
	Sometimes true	0.62	0.51	0.03	0.30	1.53	54.17	35.41	3.81	2.49	0.07
	Very true	0.07	0.04	0.00	0.50	1.67	695.12	416.63	0.07	0.04	0.00
**Sleep less than most kids**	Not true	0.03	0.98	0.86	0.32	0.00	0.00	9.25	0.06	116.46	12.59
	Sometimes true	0.94	0.00	0.10	0.02	151275.19	134.66	0.00	615.25	0.00	4.57
	Very true	0.04	0.02	0.04	0.66	1.98	0.79	0.40	0.02	0.01	0.02
**Sleep more than most kids**	Not true	0.49	0.30	0.67	0.65	2.22	0.47	0.21	0.52	0.23	1.11
	Sometimes true	0.47	0.49	0.17	0.09	0.92	4.30	4.67	8.89	9.68	2.07
	Very true	0.05	0.21	0.16	0.27	0.18	0.26	1.40	0.14	0.75	0.54
**Trouble sleeping**	Not true	0.17	0.50	1.00	0.17	0.21	0.00	0.00	1.02	4.96	1224.37
	Sometimes true	0.62	0.49	0.00	0.25	1.73	411.48	238.28	4.91	2.84	0.01
	Very true	0.21	0.01	0.00	0.58	28.39	2578.36	90.81	0.19	0.01	0.00

Note. N = 1,041. Cells are highlighted in blue to indicate probabilities that are generally different than chance (i.e., probability > 0.70 or < 0.30) or highlighted in green to indicate adequate class separation (i.e., >5 or < 0.2).

Figure S3. Item probability plot for the 4-class model (N = 1,041).

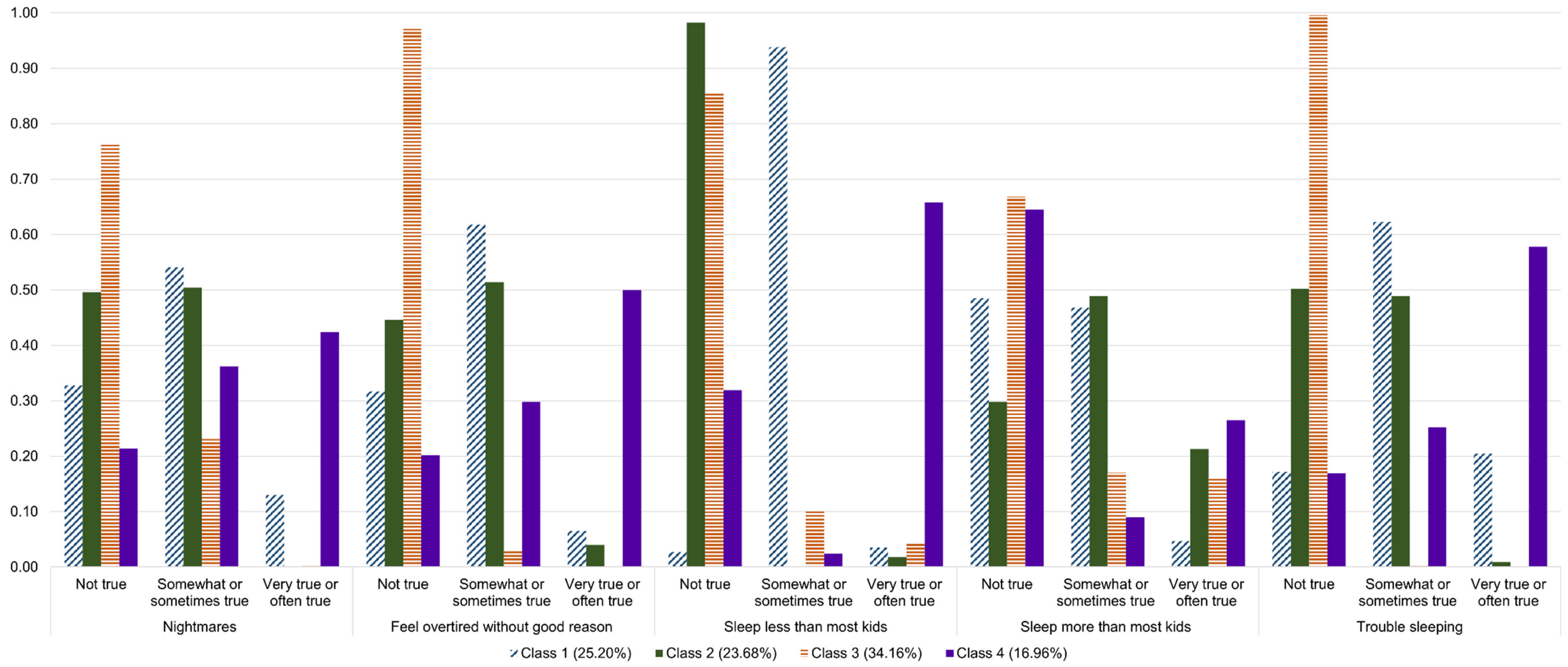

Supplemental Table S5. Classification quality of the enumerated 2-class and 3-class models with binary response scale.

k	Mean	mcaP	AvePP	OCC	Entropy
**2-class model**					
1	0.56	0.55	0.94	12.57	0.731
2	0.44	0.45	0.90	11.62	
**3-class model**					
1	0.35	0.38	0.86	11.40	0.70
2	0.17	0.16	0.71	11.77	
3	0.47	0.47	0.91	10.60	

Note. N = 1,041. AvePP = average posterior probabilities, values > 0.70 are acceptable; OCC = odds of correct classification, values >5 support adequate class separation.

Supplemental Table S6. Item response probabilities for the 3-class model using binary response scale.

Sleep item	Probability of endorsing sleep item			Class separation		
	Class 1 (15.55%)	Class 2 (46.53%)	Class 3 (37.92%)	1 vs. 2	1 vs. 3	2 vs. 3
Nightmares	0.45	0.73	0.26	0.30	2.31	7.58
Feel overtired without good reason	0.64	0.71	0.08	0.71	20.94	29.49
Sleep less than most kids	0.13	0.75	0.15	0.05	0.84	17.64
Sleep more than most kids	1.00	0.41	0.32	146.31	212.50	1.45
Trouble sleeping	0.41	0.87	0.03	0.10	19.42	193.56

Note. N = 1,041. Cells are highlighted in blue to indicate probabilities that are generally different than chance (i.e., probability > 0.70 or < 0.30) or highlighted in green to indicate adequate class separation (i.e., >5 or < 0.2).
